# Underrepresented in Medicine and Gender Trends in Ophthalmology: A Retrospective Analysis

**DOI:** 10.1177/23821205261478423

**Published:** 2026-08-11

**Authors:** Audrey Schulze, Joshua Wang, Avinash Singh, Misha Syed, Praveena Gupta

**Affiliations:** 1 John Sealy School of Medicine, 12338 University of Texas Medical Branch, Galveston, TX, USA; 2 5225 Mercer University, Macon, GA, USA; 3 Department of Ophthalmology & Visual Sciences, 12338 University of Texas Medical Branch, Galveston, TX, USA

**Keywords:** ophthalmology residency trends, underrepresented in medicine, ophthalmology education, women in ophthalmology

## Abstract

Representation in the physician workforce is essential for equitable patient care, yet trends in underrepresented in medicine (URIM) and female representation in ophthalmology relative to other specialties remain unclear. We conducted a population-based retrospective cross-sectional analysis from 2011 to 2024 to evaluate trends in URIM and female representation in ophthalmology compared with other surgical and nonsurgical residency programs. We also compared these trends among applicants, matched participants, and active residents within ophthalmology. Participants included ophthalmology residents in ACGME-accredited programs (2011–2024) and applicants and matched participants in the San Francisco Match (2016–2024). Primary outcomes were the proportions of URIM and female applicants and residents and changes over time. Data included 5,664 ophthalmology applicants (3,734 matched) for race/ethnicity and 6,206 applicants (4,420 matched) for gender from 2016–2024, and more than 1.27 million residents for race/ethnicity and 1.4 million for gender from 2011–2024. URIM representation increased significantly among applicants from 8.75% to 24.3% (absolute increase 15.6 percentage points, 95% CI 12.06–19.08) and among matched participants from 6.61% to 30.26% (23.6 percentage points, 95% CI 19.1–28.2). Among residents, URIM representation rose from 7.3% to 13.4% (6.1 percentage points, 95% CI 3.92–8.28). Female applicants increased from 40.0% to 45.2%, and matched female participants from 41.4% to 47.9%. However, female representation among ophthalmology residents declined slightly from 42.4% to 40.6% (−1.8 percentage points, 95% CI −5.5 to −1.9), while increasing significantly in other surgical specialties (+8.6 percentage points, 95% CI 7.7–9.5). Trend analyses confirmed statistical significance (all p < 0.001). Ophthalmology has made meaningful gains in URIM representation across the training pipeline, but improvements in gender representation have not translated into sustained increases among active residents, underscoring the need for longitudinal, specialty-specific strategies beyond recruitment alone.

## Introduction

Bias in ophthalmology residency matching remains a critical issue that affects diversity in the specialty, a field that has historically lagged behind other specialties in equitable representation.^1-3^ Evidence demonstrates that healthcare workforce diversity is fundamental to optimizing patient outcomes, eliminating health disparities, and ensuring culturally sensitive care practices.^4-7^ A workforce that reflects the diverse populations it serves can enhance patient trust, communication, and adherence to treatment plans.^8^ In medical education, increasing diversity in residency programs is a key step toward achieving a more representative physician workforce. Recognizing this, various initiatives have aimed to enhance representation across both surgical and nonsurgical specialties, including ophthalmology.^9,10^ However, progress has been inconsistent, with some fields seeing greater success than others in recruiting and retaining underrepresented in medicine (URIM) and female trainees.^1^

Ophthalmology, like many other surgical specialties, has faced challenges in achieving gender and racial representation that mirrors the general population it serves.^1^ While there have been modest improvements in URIM and female representation over time, disparities persist.^2,11^ Compared to other medical and surgical specialties, ophthalmology has historically lagged in female and URIM representation, raising concerns about equitable access to opportunities in the field.^3^ Factors such as limited early exposure, mentorship barriers, and systemic biases have contributed to these ongoing challenges.^12-16^ Addressing these biases in the ophthalmology match process is critical to improving diversity and equity in the specialty.

Current studies have shown slight increases in URIM applicants and matches, but these gains remain smaller than those seen in other specialties.^1^ Similarly, despite overall national growth in female representation across medicine, ophthalmology has experienced periods of stagnation or decline in female resident proportions.^2,11^ Addressing this knowledge gap requires a comprehensive comparison of URIM and female representation trends across surgical and nonsurgical specialties to clarify whether ophthalmology’s diversity challenges are specialty-specific or manifestations of broader systemic issues. By examining the entire residency pipeline, including applicants, matched participants, and active residents, areas of attrition and inequity can be more precisely identified. This approach provides an evidence base to guide targeted interventions aimed at strengthening inclusivity in ophthalmology training and promoting a more representative physician workforce.^4-7,9,10,12-16^

## Methods

### Study Design and Reporting Guidelines

We conducted a retrospective cross-sectional analysis of publicly available aggregate data from Accreditation Council for Graduate Medical Education (ACGME) Data Resource Books and Association of University Professors of Ophthalmology (AUPO)/San Francisco Match (SF Match) ophthalmology residency reports. The study evaluated temporal trends in underrepresented in medicine (URIM) and female representation in ophthalmology compared with other surgical and nonsurgical specialties. The reporting of this study conforms to the Strengthening the Reporting of Observational Studies in Epidemiology (STROBE) statement^17^ [Supplementary File 1].

### Definition of URIM and Gender-Related Variables

Consistent with the Association of American Medical Colleges (AAMC) definition, underrepresented in medicine (URIM) was used to describe racial and ethnic populations that are underrepresented in the U.S. medical profession relative to their numbers in the general population.^18^ Race and ethnicity are socially constructed categories that reflect sociopolitical classification and lived experience rather than innate biologic differences; therefore, these variables were used only to evaluate representation within the ophthalmology training pipeline. In this analysis, URIM included individuals self-identifying in the available ACGME and AUPO/SF Match categories as Black or African American, Hispanic/Latino, American Indian or Alaska Native, and Native Hawaiian or Other Pacific Islander, where separately available. Non-URIM included individuals recorded as White or Asian. Individuals recorded as more than one race, other, unknown, or declined to state were excluded from the primary binary proportion calculations because aggregate datasets did not permit consistent recoding across years.

Because the source datasets reported demographic information using the binary categories “female” and “male” under gender or sex-related variables and did not consistently provide separate measures of sex assigned at birth, gender identity, or nonbinary identity, analyses of gender representation were limited to individuals recorded as female or male. We use “female” when referring to the category label available in the source data and “women” when discussing broader implications for gender representation in ophthalmology. Individuals recorded as other, nonbinary where available, or declined to state were excluded from binary proportion calculations.

### Data Analysis

#### Current Resident Trends

Accreditation Council for Graduate Medical Education (ACGME) reports from 2011-2024 were accessed online through publicly available *Data Resource Books* published annually.^19-31^ Only pipeline programs were included, which the ACGME defines as residency programs that lead to a primary board certification. Distinctions of surgical and nonsurgical specialties were categorized within these reports and were subsequently used in this study.

Besides ophthalmology, other surgical specialties included were neurological surgery, obstetrics and gynecology (OBGYN), orthopedics, otolaryngology, integrated plastics, general surgery, integrated vascular, integrated thoracic, and urology. The nonsurgical specialties included anesthesiology, dermatology, emergency medicine, family medicine, internal medicine, medical genetics and genomics, neurology, child neurology, nuclear medicine, osteopathic neuromusculoskeletal medicine, integrated interventional radiology, pathology, pediatrics, physical medicine and rehabilitation, public health/preventative medicine, aerospace medicine, occupational/environmental medicine, psychiatry, radiation oncology, radiology, and internal medicine/pediatrics. Data on the number of residents in each specialty, along with their demographics for race/ethnicity and gender, were also collected through self-reported surveys.

Using the collected data, we sought to look at trends in the proportion of female to male residents and underrepresented in medicine (URIM) to non-URIM. Female proportion was calculated by dividing the number of residents who selected female over the number of residents who selected male for each year. This proportion was then calculated for the groups of nonsurgical, surgical, ophthalmology, and all residencies annually. Those who declined to state or selected “other” were excluded from these numbers. URIM representation was compared to non-URIM, which is defined as White and Asian. The proportion of URIM to non-URIM was calculated with the same technique as the female proportion for each group (nonsurgical, surgical, ophthalmology, all) for each year of the study. Those who selected “2+”, “other”, or declined to state were excluded from these counts.

#### Applicant and Match Trends

Data on applicants to the San Francisco Match (SF Match) that ophthalmology residencies participate in was collected from publicly available data from the Association of University Professors of Ophthalmology (AUPO) Gender and Ethnicity Data books for the years 2016-2024.^32,33^ This resource publishes information on self-reported demographics and statistics of those who registered for, applied to, and successfully matched into ophthalmology residencies through SF Match. This resource is published every 4 years, and data before 2016 is not available. We organized data from those who completed the application for ophthalmology residency (applicants) and those who successfully matched (matched participants).

Using this collected data, we sought to look at trends in the proportion of female to male applicants and matched participants and underrepresented in medicine (URIM) to non-URIM. Female proportion was calculated by dividing the number of students who selected female over the number of residents who selected male for each year. Those who declined to state or selected “other” were excluded from these numbers. URIM representation was compared to non-URIM, which is defined as White and Asian. The proportion of URIM to non-URIM was calculated with the same technique as the female proportion for applicants and matched participants for each year of the study. Those who selected “2+”, “other”, or declined to state were excluded from these counts to decrease inconsistencies in grouping.

Data was given in percent values, and the numerical values were estimated with the given totals for the number of students in each category each year.

### Statistical Analysis

A simple linear regression analysis and Cochran-Armitage test for proportion and trend were performed for each variable studied over time. This was performed using Python 3.9.16. To quantify the magnitude of change, we calculated percentage point changes with 95% confidence intervals using two-sample z-tests for proportion comparisons. Both absolute (percentage point) and relative (percent) changes were computed to provide a comprehensive assessment of trends. Effect sizes were reported as annual percentage point changes derived from linear regression slopes. A two-sided p-value <0.05 was considered statistically significant.

### Statement of Ethics

This study used publicly available, aggregate, de-identified data and did not involve direct interaction with human participants. The University of Texas Medical Branch Institutional Review Board deemed this study exempt on August 9, 2024. Because the study did not include patient-level data or identifiable participant information, informed consent was not required.

## Results

### Ophthalmology Match Applicants

#### URIM Trend Analysis

**Figure 1.**
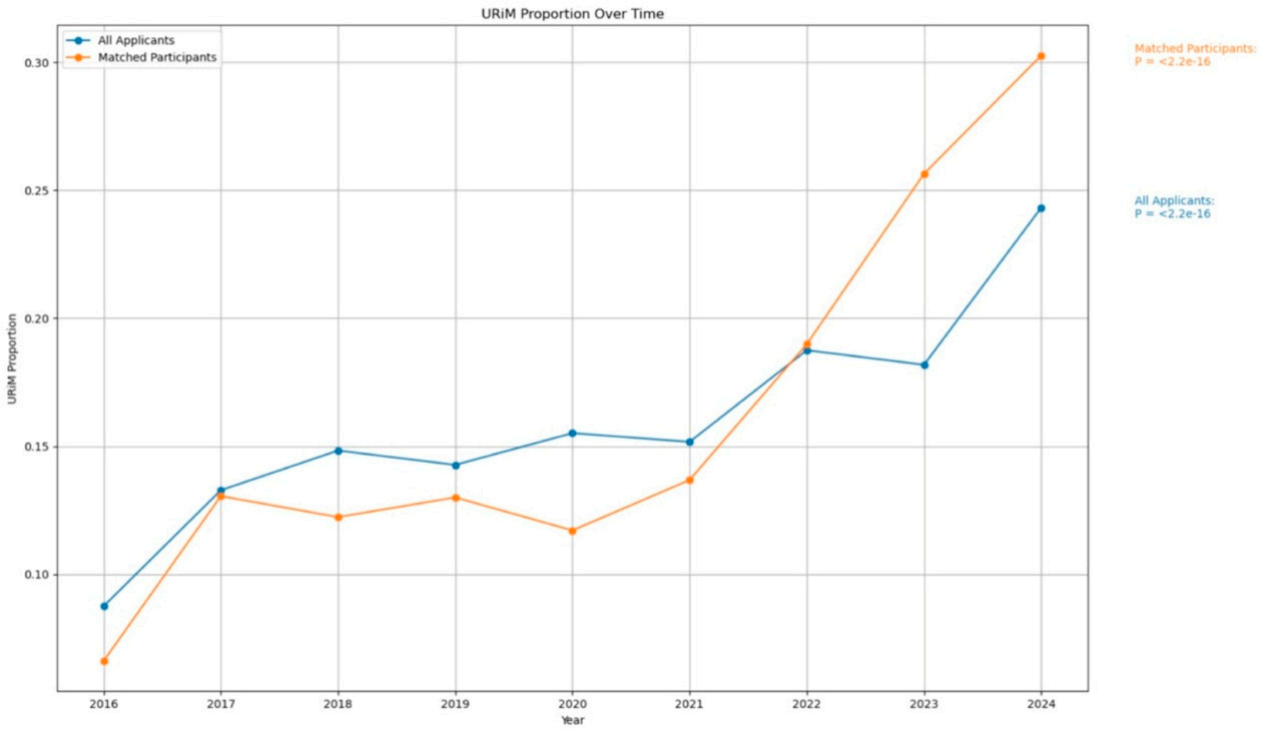
Trend of the proportion of URIM applicants and matched participants from 2016 to 2024

Data on race and ethnicity were obtained from 5,664 applicants and 3,734 matched participants. Among applicants, URIM representation increased from 8.75% in 2016 to 24.3% in 2024 (+15.6 percentage points, 95% CI: 12.06–19.08), reflecting a significant upward trend over time (Cochran–Armitage test, p < 0.001) and an estimated annual increase of 1.43 percentage points. A similar pattern was observed among matched participants, with URIM representation rising from 6.61% to 30.26% during the study period (+23.6 percentage points, 95% CI: 19.1–28.2), corresponding to a significant temporal trend (Cochran–Armitage test, p < 0.001) and an annual increase of 2.44 percentage points. Figure 1 depicts trends in URIM representation among ophthalmology applicants and matched participants from 2016 through 2024.

#### Gender Trend Analysis

**Figure 2.**
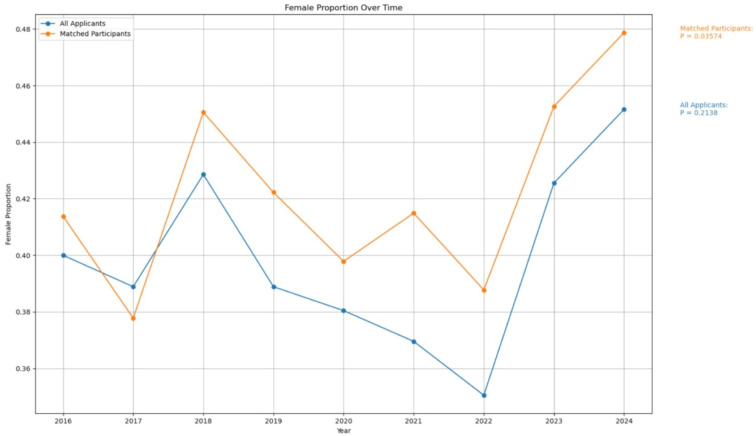
Trend of the proportion of female applicants and matched participants from 2016 to 2024

Data on gender were obtained from 6,206 applicants and 4,420 matched participants. Among applicants, the proportion of females increased from 40.0% in 2016 to 45.2% in 2024 (+5.2 percentage points, 95% CI: 0.31–10.01), reflecting a non-significant upward trend over time (Cochran–Armitage test, p = 0.064) and an estimated annual increase of 0.23 percentage points. Similarly, female representation among matched participants rose from 41.4% to 47.9% during the study period (+6.5 percentage points, 95% CI: 0.29–12.7), corresponding to a non-significant temporal trend (Cochran–Armitage test, p = 0.060) and an annual increase of 0.59 percentage points. Figure 2 illustrates trends in female representation among ophthalmology applicants and matched participants from 2016 through 2024.

### Ophthalmology Residents

#### URIM Trend Analysis

Data on race and ethnicity was obtained from over 1.27 million residents across all years. There were 17,739 ophthalmology residents, 287,601 surgical residents excluding ophthalmology, and 966,201 nonsurgical residents.

**Figure 3.**
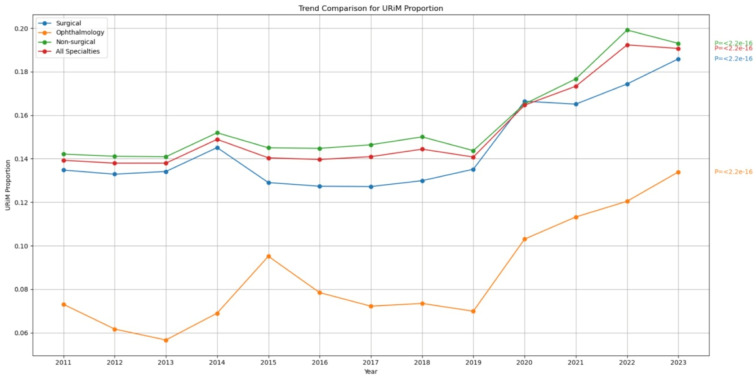
Trend of the proportion of URIM residents across all groups analyzed from 2011-12 to 2023-24

URIM representation increased significantly across all categories. In ophthalmology, URIM representation rose from 7.3% in 2011 to 13.4% in 2024 (+6.1 percentage points, 95% CI: 3.92–8.28; χ^2^ = 84.2; p < 0.001), corresponding to an 84% relative increase and an estimated annual increase of 0.51 percentage points. Other surgical specialties experienced a similar upward trend, with URIM representation increasing from 13.5% to 18.6% (+5.1 percentage points, 95% CI: 4.4–5.8; χ^2^ = 533; p < 0.001), representing a 38% relative increase and an annual increase of 0.39 percentage points. Trends in URIM representation among ophthalmology residents did not differ significantly from those observed in other surgical specialties (t = −0.78; p = 0.45). Figure 3 depicts URIM representation across all groups from 2011–2012 through 2023–2024.

#### Gender Trend Analysis

Data on gender was obtained from 1,403,800 residents across all years. There were 19,549 ophthalmology residents, 307,353 surgical residents excluding ophthalmology, and 1,076,898 nonsurgical residents.

**Figure 4.**
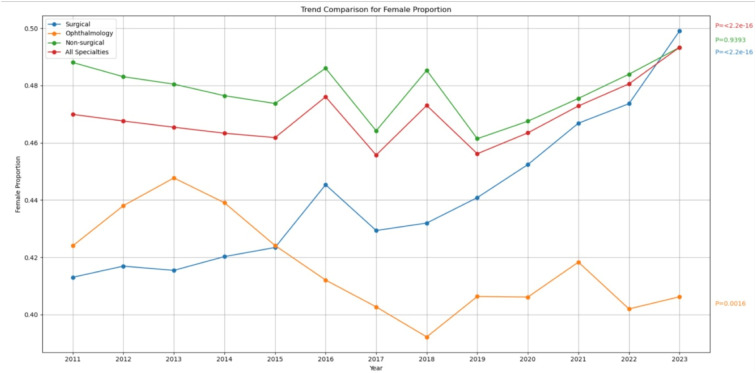
Trend of the proportion of female residents across all groups analyzed from 2011-12 to 2023-24

Among ophthalmology residents, the proportion of women declined from 42.4% in 2011 to 40.6% in 2024 (−1.8 percentage points; 95% CI, −5.5 to 1.9; two-proportion comparison p = .309). Across the full study period, however, trend testing demonstrated a statistically significant downward trend (χ^2^ = 9.93, df = 1, p = .002), corresponding to an estimated annual decrease of 0.15 percentage points. In contrast, female representation in other surgical specialties increased substantially, rising from 41.3% to 49.9% over the same period (+8.6 percentage points, 95% CI: 7.7–9.5; χ^2^ = 699; p < 0.001), corresponding to an annual increase of 0.67 percentage points. The trajectory of female representation in ophthalmology differed significantly from that of other surgical specialties (t = 7.6; p < 0.001). Figure 4 illustrates these trends in female resident representation across all groups from 2011–2012 through 2023–2024.

## Discussion

This study demonstrates that ophthalmology has made measurable gains in URIM representation across the residency training pipeline, particularly among applicants and matched participants. Between 2016 and 2024, URIM representation increased from 8.8% to 24.3% among applicants and from 6.6% to 30.3% among matched participants. Among active residents, URIM representation also increased from 7.3% in 2011 to 13.4% in 2024. These findings suggest that recent efforts to improve recruitment and selection may be associated with meaningful changes in the demographic composition of ophthalmology trainees. However, despite this progress, ophthalmology continues to have lower absolute URIM representation than many comparison groups, indicating that gains in representation have not yet eliminated longstanding underrepresentation within the specialty.

This analysis adds to prior literature by examining multiple stages of the ophthalmology training pathway rather than evaluating applicants, matched participants, or residents in isolation. Prior work has demonstrated demographic differences in ophthalmology match outcomes and persistent underrepresentation within the field.^2,10,34^ In particular, Mehta et al recently examined ophthalmology match rates by applicant sex, race, and ethnicity, highlighting the importance of evaluating not only broad demographic categories but also how overlapping identities may shape match outcomes.^34^ By combining applicant, match, and resident-level data, our findings suggest that the ophthalmology pipeline has changed unevenly over time. URIM representation has improved across all measured stages, while gains in female representation among applicants and matched participants have not translated into sustained increases among active residents. This distinction is important because recruitment-focused improvements alone may not be sufficient if retention, training climate, mentorship, or professional development barriers persist after the match.

The increase in URIM representation may reflect several overlapping changes within ophthalmology and medical education more broadly. These include greater attention to holistic review, increased awareness of structural barriers in residency selection, targeted mentorship and pipeline programs, scholarships and away rotation support, and bias-reduction practices within residency recruitment.^9,10^ National conversations about diversity, equity, and inclusion may also have prompted programs to examine selection criteria, outreach strategies, and applicant evaluation processes more critically. Because our study used aggregate data, we cannot determine which specific interventions contributed most directly to these trends. However, the temporal increase in URIM representation among applicants, matched participants, and residents suggests that continued investment in structured, evidence-based recruitment strategies remains important.

In contrast, female representation in ophthalmology showed a more concerning pattern. Female applicants increased from 40.0% to 45.2%, and matched female participants increased from 41.4% to 47.9% between 2016 and 2024. However, the proportion of female ophthalmology residents declined from 42.4% in 2011 to 40.6% in 2024, while female representation in other surgical specialties increased substantially during the same period. This divergence suggests a possible mismatch between recruitment into ophthalmology and sustained representation during residency. Further work is needed to determine whether this pattern reflects differences in applicant behavior, match outcomes, residency attrition, reporting practices, program culture, or broader structural barriers affecting women in ophthalmology.

Several factors may contribute to this pattern. Prior studies have described gender disparities in mentorship, sponsorship, leadership visibility, and career advancement within ophthalmology.^13,14^ Women ophthalmologists have also reported discrimination, workplace challenges, and unmet family or personal time needs, all of which may influence career satisfaction and retention.^15^ Early perceptions of ophthalmology, including limited exposure to the specialty, assumptions about procedural opportunities, and concerns about work-life integration, may also shape whether medical students view ophthalmology as an accessible and supportive career path.^12^ These findings suggest that interventions to improve gender representation should extend beyond recruitment and address the training environment, mentorship structures, parental leave culture, sponsorship, and pathways to leadership.

The inability to evaluate intersectionality is an important consideration. The available aggregate datasets did not allow us to examine individuals who hold multiple marginalized identities, such as women who are also URIM. This limitation is particularly relevant in light of recent work by Mehta et al, which evaluated ophthalmology residency match rates at the overlap of sex, race, and ethnicity and underscored the importance of intersectional analyses in understanding representation within the ophthalmology match.^34^ As a result, our analysis may obscure important differences between female URIM trainees, female non-URIM trainees, male URIM trainees, and male non-URIM trainees. Intersectional analyses are especially important because barriers related to race, ethnicity, and gender may not operate independently. Future studies using individual-level, de-identified longitudinal data should evaluate how overlapping identities influence application patterns, match success, residency experiences, attrition, mentorship access, and career progression within ophthalmology.

The implications of these findings extend beyond numerical representation. A diverse physician workforce is essential for improving patient trust, communication, cultural humility, and care for underserved communities.^4-8^ In ophthalmology, where disparities in access to screening, treatment, and surgical care remain persistent, a workforce that better reflects the population it serves may help strengthen patient-centered care and reduce inequities. At the same time, representation within residency is closely tied to future faculty, leadership, and mentorship pipelines. Without sustained attention to both recruitment and retention, ophthalmology risks limiting the diversity of future educators, researchers, program leaders, and department chairs.

### Limitations

This study has several limitations. First, analyses relied on binary categorizations of gender and URIM status due to the structure of publicly available aggregate datasets. This approach does not capture the full complexity of race, ethnicity, sex, or gender identity and may obscure important within-group differences. Dichotomizing race and ethnicity into URIM versus non-URIM limits evaluation of individual groups and prevents assessment of multiracial identities, Middle Eastern or North African identities, and other categories not consistently reported across datasets. Similarly, the gender analysis was limited to individuals recorded as female or male and could not assess nonbinary or transgender trainees.

Second, this dataset did not allow intersectional analyses because applicant, matched-participant, and resident data were not cross-tabulated by both race/ethnicity and gender. As a result, we could not determine whether trends differed among URIM females, URIM men, non-URIM females, or non-URIM men. Given the relevance of intersectionality to representation and retention, future studies should use individual-level deidentified data to examine how overlapping identities shape ophthalmology application, match, and training outcomes.

Third, changes in racial and ethnic reporting practices over time may have affected longitudinal comparisons. In ACGME reports from 2011 to 2019, Pacific Islander individuals were grouped with Asians, which may have inflated the non-URIM category during these years. Beginning in 2019, Pacific Islander individuals were categorized with American Indian or Alaska Native groups, which are classified as URIM in this analysis. Although unavoidable given the data structure, this shift introduces potential inconsistency across study years.

Fourth, applicant and match-level analyses relied on SF Match summary reports, which did not provide raw counts for demographic subgroups. As a result, numerical estimates were derived from reported percentages and total applicant or matched counts. Because these percentages were rounded, minor imprecision in reconstructed subgroup sizes is possible, particularly for smaller categories.

Fifth, the number of ophthalmology residents and applicants is substantially smaller than that of aggregated surgical and nonsurgical comparison groups. Although ophthalmology-specific trends were internally consistent and statistically evaluated, smaller sample sizes may reduce power to detect subtle changes and increase variability in year-to-year estimates.

Finally, this study is descriptive in nature and based on aggregate, publicly available data. While we observed clear temporal patterns in recruitment and resident composition, these findings cannot determine why these changes occurred or identify the specific factors contributing to post-match attrition, particularly among women. Addressing these questions will require future work using individual-level longitudinal data and qualitative approaches to better understand the experiences that influence retention during ophthalmology training.

## Conclusions

Ophthalmology’s targeted initiatives for growing a more representative workforce have yielded substantial gains in racial and ethnic representation at each stage of the residency pipeline. URIM proportions among applicants, matched participants, and active residents have more than doubled over the study period. Despite incremental improvements in the proportion of female applicants and matched participants, the percentage of women in active residency positions has declined. This divergence indicates that recruitment efforts alone are insufficient to ensure sustained gender equity. Sustaining both URIM and gender diversity will require pairing holistic review and scholarships with formal mentorship and sponsorship for women, regular bias-awareness training for selection committees, early-exposure programs, and ongoing evaluation of outcomes to ensure that recruitment gains translate into a workforce reflective of the patients ophthalmology serves.

## Supplemental Material

Supplemental Material - Underrepresented in Medicine and Gender Trends in Ophthalmology: A Retrospective Analysis Supplemental Material for Underrepresented in Medicine and Gender Trends in Ophthalmology: A Retrospective Analysis by Audrey Schulze, Joshua Wang, Avinash Singh, Misha Syed, Praveena Gupta in Journal of Medical Education and Curricular Development.
