# Supplementary material for: Underrepresented in Medicine and Gender Trends in Ophthalmology: A Retrospective Analysis
Source: J Med Educ Curric Dev. 2026 Aug 11;13:23821205261478423. doi: 10.1177/23821205261478423 (PMC13462495; doi:10.1177/23821205261478423)
Supplement: Supplementary file 1 [file sj-pdf-1-mde-10.1177_23821205261478423.pdf]

Supplementary File 1. STROBE Checklist for Cross-Sectional Studies

Manuscript: Underrepresented in Medicine and Gender Trends in Ophthalmology: A Retrospective Analysis

Study design: Retrospective cross-sectional analysis using publicly available aggregate ACGME and AUPO/SF Match data.

Section	Item	Checklist item	Reported location / notes
Title and abstract	1(a)	Indicate the study design with a commonly used term in the title or abstract.	Title and abstract identify a retrospective analysis/cross-sectional analysis.
Title and abstract	1(b)	Provide an informative and balanced summary of what was done and what was found.	Abstract summarizes data sources, timeframe, outcomes, main trends, and conclusion.
Introduction	2	Explain the scientific background and rationale for the investigation.	Introduction describes workforce diversity, ophthalmology representation gaps, and prior literature.
Introduction	3	State specific objectives, including any prespecified hypotheses.	Objective stated as evaluating URIM and female representation trends in ophthalmology compared with surgical and nonsurgical specialties and across the pipeline.
Methods	4	Present key elements of study design early in the paper.	Methods describe retrospective cross-sectional design using annual aggregate data. Methods includes a STROBE statement.
Methods	5	Describe the setting, locations, and relevant dates, including periods of recruitment, exposure, follow-up, and data collection.	Methods list ACGME 2011-2024 resident data and AUPO/SF Match 2016-2024 applicant and match data.
Methods	6(a)	Give eligibility criteria and the sources and methods of selection of participants.	Methods specify inclusion of ACGME pipeline residency programs and ophthalmology SF Match applicants/matched participants.
Methods	7	Clearly define all outcomes, exposures, predictors, potential confounders, and effect modifiers.	Primary outcomes are proportions recorded as URIM and female among applicants, matched participants, and residents. Methods define URIM and source-data gender terminology.
Methods	8	For each variable of interest, give sources of data and details of methods of assessment.	Methods specify self-reported demographic data from ACGME reports and AUPO/SF Match data books.
Methods	9	Describe any efforts to address potential sources of bias.	Methods and Limitations explain exclusions of declined/other/multiracial categories due to inconsistent aggregate reporting and acknowledge reporting changes over time.
Methods	10	Explain how the study size was arrived at.	Study size determined by all eligible publicly available aggregate records during study periods; Results report total applicant, matched participant, and resident counts.
Methods	11	Explain how quantitative variables were handled in the analyses.	Methods describe annual proportion calculations, absolute and relative changes, and annual percentage point changes. Methods clarify denominators.
Methods	12(a)	Describe all statistical methods, including those used to control for confounding.	Methods describe simple linear regression, Cochran-Armitage tests, two-sample z-tests for proportions, confidence intervals, and p-value threshold.
Methods	12(b)	Describe any methods used to examine subgroups and interactions.	Not applicable for individual-level subgroup/intersectional analyses because source data are aggregate. Limitations explicitly state this.
Methods	12(c)	Explain how missing data were addressed.	Individuals with declined/other/unknown categories were excluded from binary proportion calculations; Methods and Limitations state this explicitly.
Methods	12(d)	If applicable, describe analytical methods taking account of sampling strategy.	Not applicable; study used aggregate annual census-style public reports rather than sampled participant-level data.
Methods	12(e)	Describe any sensitivity analyses.	No sensitivity analyses reported. Consider adding “No sensitivity analyses were performed” if desired.
Results	13(a)	Report numbers of individuals at each stage of study.	Results report applicant, matched participant, and resident totals for race/ethnicity and gender analyses.
Results	13(b)	Give reasons for non-participation at each stage.	Not applicable; analysis used aggregate public reports. Excluded demographic categories are described in Methods/Limitations.
Results	13(c)	Consider use of a flow diagram.	Not used; not necessary for aggregate annual public datasets, but could be noted as not applicable.
Results	14(a)	Give characteristics of study participants.	Results and figures provide annual demographic proportions among applicants, matched participants, and residents.
Results	14(b)	Indicate number of participants with missing data for each variable.	The source reports did not provide enough raw counts for all missing categories consistently; Methods and Limitations explain exclusions.
Results	14(c)	Summarize follow-up time.	Not applicable to cross-sectional annual aggregate trend analysis.
Results	15	Report numbers of outcome events or summary measures.	Results report proportions, absolute percentage point changes, confidence intervals, p-values, and annual changes.

Results	16(a)	Give unadjusted estimates and, if applicable, confounder-adjusted estimates and precision.	Unadjusted proportions and changes with 95% confidence intervals are reported; adjusted estimates are not applicable to aggregate descriptive data.
Results	16(b)	Report category boundaries when continuous variables were categorized.	Not applicable; demographic categories were defined by source datasets and Methods.
Results	16(c)	If relevant, consider translating estimates of relative risk into absolute risk.	Absolute percentage point changes and relative changes are reported.
Results	17	Report other analyses done, such as subgroup and sensitivity analyses.	Trend analyses are reported. Intersectional subgroup analyses and sensitivity analyses were not possible with aggregate data; Limitations state this.
Discussion	18	Summarize key results with reference to study objectives.	Discussion summarizes URIM gains and gender divergence.
Discussion	19	Discuss limitations, taking into account sources of potential bias or imprecision.	Limitations address binary categories, reporting changes, reconstructed counts, smaller ophthalmology sample, and descriptive nature. Limitations expand race/gender and intersectionality.
Discussion	20	Give a cautious overall interpretation of results considering limitations and other evidence.	Discussion and Conclusion interpret findings cautiously and connect to prior literature.
Discussion	21	Discuss generalisability of the study results.	Generalizability is to U.S. ophthalmology residency pipeline and ACGME-accredited programs represented in public reports.
Other information	22	Give the source of funding and role of funders.	Funding statement identifies Robertson Poth Foundation support.
